# Migraine Treatment in the Emergency Department: Alternatives to Opioids and their Effectiveness in Relieving Migraines and Reducing Treatment Times

**DOI:** 10.7759/cureus.2439

**Published:** 2018-04-06

**Authors:** Haley Dodson, Jay Bhula, Sven Eriksson, Khoa Nguyen

**Affiliations:** 1 College of Medicine, University of Central Florida, Orlando, USA

**Keywords:** migraine, opioids, d2 receptor antagonist, metoclopramide, prochlorperazine

## Abstract

The objective of this literature review is to evaluate the efficacy of opioids for the treatment of headaches, particularly migraines, in the emergency department (ED). Despite safer alternatives, opiates are routinely used as an abortive treatment for migraine headaches. The studies reviewed demonstrate that opiates are less effective in terminating acute headaches and result in prolonged ED visits. Dopamine receptor antagonists, such as metoclopramide and prochlorperazine, were the most efficacious in terminating migraines in the studies examined.

## Introduction and background

Migraine headache is the most common primary headache disorder that patients seek medical treatment for in the emergency department (ED), accounting for at least 1.2 million visits to the ED each year [1]. Traditionally, patients diagnosed with a migraine presented with severe, throbbing, unilateral headache lasting between four and 72 hours [2]. Costs associated with migraine treatment continue to increase with annual costs estimated at $3.2 billion for outpatient visits, $700 million for emergency room (ER) visits, and $375 million for inpatient hospitalizations [3]. Not only is there a large economic impact on patients who suffer from migraines, but there is a substantial psychosocial effect as well. Over 70% of headache sufferers report an impairment of interpersonal relationships and 78% of regular activities are limited during migraine attacks [1]. Despite recent advances in migraine research, establishing an effective medical treatment for migraines has thus far been a challenge due to the incomplete understanding of the pathophysiology [4].

Opioids traditionally have been at the forefront of abortive treatment for migraines in the ED, with morphine being the most commonly used analgesic [5]. Drug-seeking patients seem to know this, making headache the second most common symptom after back pain for drug seekers [6]. However, since 1999, overdose deaths involving prescription opioids have quadrupled [6]. Today, 40% of opioid overdose deaths involve prescription opioids [7]. There has been a nationwide push towards reducing the amount of opioids prescribed. Emergency medicine physicians have had the most substantial reduction in opioid-prescribing rates with an 8.9% reduction in opioid prescriptions from 2007 to 2012 [8]. Despite these positive trends, opioid-overdose related deaths still account for over 40% of drug overdose deaths, highlighting the need to find alternative methods for migraine management [9].

Dopamine receptor antagonists, such as metoclopramide and prochlorperazine, show promise as an effective treatment for migraine headaches [10]. While still incompletely understood, it is hypothesized that metoclopramide aborts migraine headaches through inhibiting trigeminovascular activation [11]. Recent studies show that metoclopramide is as effective as sumatriptans in migraine pain relief [12]. The dopamine receptor antagonist side effect profiles are much more benign than other pain management medications as well. Acute dystonic reactions are the most common side effect reported from metoclopramide and prochlorperazine, occurring in approximately one in 500 patients [13].

With headache being the fifth most common cause of emergency room visits, effective alternatives to narcotics could greatly reduce overall opioid use. The responsibility to relieve pain must be balanced by the risk to cause further harm which leads us to seek non-opioid managements for the fifth most common ED visit, a headache. Given the relative safety profile of dopamine receptor antagonists compared to opioids, it is imperative for us to investigate if metoclopramide or prochlorperazine can be an effective alternative for aborting migraine headache symptoms and sustaining pain relief.

## Review

Metoclopramide vs hydromorphone

Northwestern Memorial Hospital in Chicago, IL conducted a retrospective cohort study comparing the effects of metoclopramide to hydromorphone for the treatment of migraine headaches in the emergency department [14]. The study used patients’ self-reported pain scores to determine the efficacy of treatment. Two hundred subjects were included with 51 receiving either intravenous (IV) or intramuscular (IM) hydromorphone, 95 receiving IV metoclopramide, and 54 receiving one of several other medications. The study included patients greater than 17 years of age who met the International Classification of Diseases-9 (ICD-9) of migraine headache treated between October 2002 and March 2003. Potential study subjects were excluded if they had non-migraine etiology of their headache, fever greater than 100.5F, meningeal signs, altered mental status, focal neurological deficit, a history of antecedent trauma, pregnancy, or breastfeeding. The subjects’ age, gender, and race were recorded to assess for any potential confounding variables.

Of the 51 subjects that received hydromorphone, 48 (94.1%) were administered the medication intravenously and three (5.9%) intramuscularly (15 received 0.5 mg, 26 received 1.0 mg, seven received 2.0 mg, and three received 4.0 mg). All subjects that received metoclopramide were administered their medication intravenously (37 received 10 mg and 58 received 20 mg). Metoclopramide was found to reduce subjects’ self-reported pain scores by 3.7 points, compared to 2.3 point reduction for hydromorphone and 2.8 points for other medications, including promethazine, ondansetron, sumatriptan, ibuprofen, ketorolac, and acetaminophen. There was no difference in pain reduction between 10 mg and 20 mg doses of metoclopramide (P = .35) or between 0.5 mg, 1 mg, 2 mg, and 4 mg doses of hydromorphone (P = .24). Metoclopramide also resulted in less use of rescue medications, faster times to discharge, with no more frequent adverse reactions [14].

The authors concluded that 10 to 20 mg of metoclopramide IV is safe and effective for the initial treatment of migraine in the ED and may be more effective than hydromorphone. The study did not mention if patients self-administered medication prior to their arrival in the emergency department which may have affected the results. Due to the retrospective nature of the study, there was no long-term follow-up for any of the subjects. As a result, the effect of metoclopramide versus hydromorphone for any substantial period after the subjects’ emergency department visits could not be assessed.

Prochlorperazine vs hydromorphone

Prochlorperazine, another dopamine receptor antagonist, is being used as an alternative to opioids for the treatment of migraines. A double blind, randomized control trial of 126 patients who presented the ED at Montefiore Medical Center in the Bronx, NY with a presenting diagnosis of migraine headache sought to compare the efficacy of prochlorperazine plus diphenhydramine to that of hydromorphone. Eligible patients met the International Classification of Headache Disorders, 3rd edition (ICHD-3) Beta criteria for moderate or severe migraine, were afebrile, ≥21 years of age, had no opioid use within the previous month, had no history of addiction to prescription or illicit opioids, and had a presentation consistent with a migraine headache. Patients were treated with either prochlorperazine plus diphenhydramine or hydromorphone and then were assessed for severity of symptoms on a subjective ‘none-mild-moderate-severe’ rating scale every hour for four hours or until discharge. Additionally, patients were assessed at 48 hours, one month, and three months using the Migrate Disability Assessment Scale. The primary outcome of sustained migraine relief was deemed successful if within two hours of medication administration there was a reduction in the severity of symptoms impairment to ‘none’ or ‘mild’ and this relief was sustained at follow-up in 48 hours. Patients who requested a second dose were considered to have failed the primary outcome, but were reassessed secondarily using the same methods after receiving the additional dose. Patients who requested additional doses were deemed outcome failures [15].

A total of 62 patients were placed into the prochlorperazine treatment arm and received prochlorperazine 10 mg IV administered over five minutes with one co-administration of diphenhydramine 25 mg. The hydromorphone arm included 64 patients and received hydromorphone 1 mg IV administered over five minutes with one normal saline placebo, for blinding purposes. The primary outcome of 48 hours sustained headache relief with one dose was successful in 60% of patients in the prochlorperazine arm, compared to only 31% of patients in the hydromorphone arm. This result represents an absolute risk reduction of 28% (95% Confidence Interval (CI): 12-45) when using prochlorperazine, which yields a number needed to treat of just four patients (95% CI: 2-9) to prevent a relapse of headache relief that would have occurred with hydromorphone. The secondary outcome of relief with one or two doses was successful in 60% of patients in the prochlorperazine arm, compared to 41% of patients in the hydromorphone arm, representing an absolute risk reduction of 19% (95% CI: 2-36), yielding a number needed to treat of six patients (95% CI: 3-52) [15].

During the data collection, the authors concluded that prochlorperazine is superior to hydromorphone for the treatment of migraine in the ED and they terminated the study early with 126 participants, when a priori analysis determined a necessary sample size of 208. This early termination, which increases the risk of a type two error, is one of several limitations in this study. One significant limitation is that history of headache was not assessed, leaving chronic migraine sufferers vs acute migraine a potentially confounding variable, affecting the generalizability of results. Another limitation is that patients were not assessed for a history of exposure to investigational medications, potentially unblinding the study if a patient is familiar enough to recognize the substance by its effects. Other limitations include the fact that there was no assessment to see if subsequent visits and treatments were just as effective. Additionally, all patients were older than 21, the average age was 32 ± 9, and 79% were female. It is possible that these results will not apply to patients who do not fit this demographic. That said, given the double-blind, randomized nature of the study structure, and the magnitude of the difference in effectiveness, this study presents sufficient evidence for the use of prochlorperazine over hydromorphone in the treatment of adult migraine headaches in the ED.

Prochlorperazine vs ketamine

Although not specific to migraines, a double blind, randomized, controlled study compared IV prochlorperazine with IV ketamine for treatment of benign headaches in the ED. The main selection criteria included patients who presented to the ED with a headache, age 18 to 65 years, temperature less than 100.4° F, diastolic blood pressure less than 104 mmHg, and normal neurological examination results. Exclusion criteria included patients who were pregnant or breastfeeding, were a prisoner, had meningeal signs, had signs of acute angle closer glaucoma, had head trauma within the previous two weeks, had a lumbar puncture within the previous two weeks, had a thunderclap onset of their headache, weighed more than 150 kg or less than 40 kg, had a known allergy to one of the study drugs, had a history of schizophrenia or bipolar disorder, had a history of intracranial hypertension, did not speak English, or had received pain medication in the ED before enrollment. The primary outcome measure was the absolute difference in pain scores between the prochlorperazine and ketamine groups measured at 60 minutes following treatment administration. The secondary outcome was the difference in pain scores at other time intervals, rate of admission, nausea score, rate of vomiting, rate of rescue medication use, rate of development of subjective restlessness, headache resolution with telephone follow-up, and patient satisfaction [16].

Pain scores were measured using a verified 100 mm visual analog scale (VAS). The headache severity scores were measured at 15, 30, 45, and 60 minutes. Time zero began immediately before administration of the study drugs. Other parameters were recorded including: vomiting, restlessness, and need for rescue medication. A trained research assistant contacted each patient 24 to 48 hours post discharge by telephone or email asking them to rate their medication satisfaction on a scale of 0 to 10 and whether they still have a headache. Between March 2016 and March 2017, 54 patients were enrolled in two Las Vegas centers. There were 29 patients assigned to receive prochlorperazine 10 mg IV along with diphenhydramine 25 mg IV and 25 patients assigned to receive ketamine 0.3 mg/kg along with ondansetron 4 mg IV [16].

The results of this study indicated that prochlorperazine was superior to ketamine in primary and secondary outcome measurements. At 60 minutes, the average VAS pain score decreased an average of 63.5 mm (95% CI: 52.7 mm to 74.3 mm) in the prochlorperazine group as compared to a decrease of 43.5 mm (95% CI: 30.2 mm to 56.8 mm) in the ketamine group. The difference in pain score reduction was statistically significant (P = 0.03). Despite three patients dropping out of the study, prochlorperazine was still superior to ketamine when the dropouts were assumed to have excellent outcomes (100% pain relief) or terrible outcomes (worsening of pain to a VAS score of 100). Vomiting was slightly decreased in the prochlorperazine group (7.1%) as compared to the ketamine group (13.0%). Eight of the 28 (28.6%) patients in the prochlorperazine group required rescue medications as compared to 11 of the 23 (47.8%) patients receiving ketamine. In the prochlorperazine group, 10.7% of the patients developed subjective restlessness as compared to 13.0% of the ketamine group patients. Among the patients who were successfully contacted 24 to 48 hours post discharge, the average satisfaction score was 8.3 out of 10 for the prochlorperazine group and 4.9 out of 10 for the ketamine group, a statistically significant difference. In the prochlorperazine group, 30% still complained of headaches 24 to 48 hours following discharge compared with 50% in the ketamine group [16].

The authors of the article concluded that prochlorperazine was superior to ketamine, specifically at the 45 and 60 minutes VAS pain score measurement and in patient satisfaction at 24 to 48 hours. A few limitations existed for this study, particularly in the limited sample size and the potential for unblinding. Due to continued concerns regarding ketamine patients experiencing extreme dysphoria, some providers refused to enroll more patients into the study. An interim analysis of the data was performed and the study was discontinued at 54 patients. In addition, many patients experienced ketamine-specific reactions such as nystagmus and confusion, potentially allowing providers to guess which medication was provided. This introduced a potential bias for providers to develop their own concerns regarding ketamine. In addition, a quarter of patients were lost to fallout, potentially introducing some degree of dropout bias and making the secondary outcome measurements at 24 to 48 hours less reliable. This article did present a strong case for providing prochlorperazine over ketamine in the treatment of headaches in the ED [16].

Non-opiates vs opiates in primary headaches

Study One

In a retrospective chart review, the electronic charts of 574 consecutive patients who visited the ED with a diagnosis of primary headache were reviewed in order to compare the patient outcomes in those treated with parenteral opiates or non-opiate recommended headache medications [17]. The primary outcome variables measured were the mean length of stay and rate of return ED visits within seven days. Consideration for inclusion in this study was given to patients aged 18 and older who presented to the Stanford Emergency Department during the study period of May 1st, 2011 to September 1st, 2012, with a chief complaint of headache or migraine and a diagnosis at discharge of headache. ICD-9 codes of headache, tension headache, migraine, or other headache syndrome were utilized to determine the discharge diagnosis of headache in this study. Subjects were excluded from analysis in this study if there was identification of any secondary cause for headache or a diagnosis that would limit treatment options, such as pregnancy, recent hemorrhage, coagulopathy, or acute or chronic renal failure [17].

Data were collected directly from the electronic medical records (EMR) clinical research database. Parenteral medications were classified as first-line recommended treatments if they were included as recommended in both recent systematic review of acute migraine treatments and also in the recent Canadian Headache Society guideline for acute migraine treatment in the ED, or were recommended as best evidence parenteral treatment of acute tension-type headaches by the European Federation of Neurological Societies (EFNS) guideline. This selection criterion excludes intranasal lidocaine, IV magnesium, droperidol, ergotamine, and promethazine. The non-opiate first-line recommended medications included in this study were prochlorperazine, metoclopramide, chlorpromazine, ketorolac, aspirin, acetaminophen, triptans, and dihydroergotamine. Opiates included morphine, dilaudid, and fentanyl [17].

The median length of stay for all subjects was 4.5 hours. However, patients who were given opiates initially had a 3.9 times higher odds of having a long ED stay (>6 hours) than those who received non-opiate first-line recommended medications (95% CI: 2.5-6.1, p < 0.001). This association remained statistically significant after adjustment for potential confounding variables.

Of the 574 subjects included in the study analysis, 69 had at least one readmission for headache during the study period and 20 had an early return visit (within seven days). Those given opiates as the initial treatment were 2.7 times more likely for an early return visit in univariate analysis (p = 0.035). In multivariable analysis, to reduce the effects of confounding variables, the association was no longer statistically significant (p = 0.15) [17].

Because this study is a retrospective chart review, there is a lack of randomization of subjects to their treatments. Additionally, there is not as much utility as a randomized control trial, in which the study is specifically designed to compare the effectiveness of opiate vs non-opiate recommended first-line medication in the treatment of primary headaches. Another limitation of this study is that the investigators grouped medications into larger, more generic categories such as opiates vs non-opiate first-line recommended medications, reducing the potential utility of this study in practice.

Study Two

Other studies have also concluded that opioid usage for migraine in the ED leads to longer ED visits. A retrospective chart review conducted from June 13, 2002 to June 16, 2003 of ED patients at an academic urban and suburban teaching hospital affiliated with University of California San Diego (UCSD) sought to compare the length of ED visits for patients treated for a primary diagnosis of migraine with opioids versus other medications. Additionally, they compared patients with only one ED visit for migraine within 12 months to patients with multiple visits, classified as 'repeaters' in this study. Charts were chosen for inclusion if the patient was being treated for a primary diagnosis of migraine as coded by ICD-9, between the ages of 18 and 65, not pregnant, not evaluated by CT or lumbar puncture, and not admitted to the inpatient service. Additionally, charts that did not report discharge times were excluded [18].

Patients were stratified based on whether or not they were ‘repeaters’, previously treated for migraines in the ED within 12 months, received opioids (meperidine, hydromorphone, morphine, fentanyl, codeine, or oxycodone) or non-opioids (ketorolac, NSAIDS, acetaminophen, butalbital/caffeine, triptans, dihydroergotamine, promethazine, prochlorperazine, metoclopramide, ondansetron, hydroxyzine, droperidol, prednisolone, valproic acid, alprazolam, diazepam, or lorazepam), were given multiple doses of opioids or a single dose, and were assessed based on minutes spent in the ED. A total of 249 visits by 189 patients met the inclusion criteria. The results showed that 90.6% of repeaters and 54.2% of non-repeaters received opioids. The ED times were significantly longer for patients treated with opioids compared to non-opioids; 142 minutes (95% CI: 124-160) vs 111 minutes (95% CI: 93-129), respectively, p = 0.015. In looking at multiple doses, 41.6% of repeaters and only 15.7% of non-repeaters required multiple doses of opioids. There was a significant difference (p = 0.003) in the time spent in the ED between patients who received multiple doses of opioids than those who received a single dose; 191 minutes (95% CI: 156-225) vs 125 minutes (95% CI: 101-149). The authors speculated that although opioids are not recommended by the guidelines for migraine treatment, perhaps ED physicians believe that they result in shorter ED visits. The authors ultimately concluded that opioids are a poor choice for reducing the length of ED visits in migraine patients [18].

There are several limitations in this study. The severity of symptoms and pre-ED medications were not considered, which could have had a significant impact on the generalizability of these results. Additionally, data were only collected from two local hospitals. There was no long-term follow-up or distinction between a repeater who visited the ED again within 24-72 hours and a repeater whose visits were months apart, confounding chronic headache with treatment failure. There was no mention of the methods of blinding used for the observer who conducted the search for charts or the abstractors who reviewed them, potentially exposing the study to selection bias. Importantly, there was no distinction within the opioid and non-opioid classes, which could be a wildly confounding variable as there is no reason to think a drug like ketorolac would have similar results to drugs like prochlorperazine or metoclopramide. Finally, the retrospective nature of this study makes a causal relationship difficult to elucidate. While it is possible that opioid’s inferiority to other medications led to longer ED stays, there are simply too many confounding variables to demonstrate that relationship exists. While there are many limitations in this study, the evidence does suggest that in addition to the fact that opioids are not recommended as first-line therapy based on patient symptom outcomes, from the healthcare system point of view, they may cause a greater burden by increasing throughput times [18].

Study Three

In another study comparing the efficacy of opioids versus non-opioid medications for the treatment of migraine headaches, Young et al. found opioids to be inferior in the treatment of acute migraine in the ED [19]. This cross-sectional study looked at three different ED settings in Connecticut, an academic medical center, a non-academic urban ED, and a community ED between January 1, 2014 and February 27, 2015 to determine the prevalence of opioid orders, rescue medications required, and length of stay [19].

Patients were identified from database maintained by d2i, formerly Emergency Medicine Business Intelligence (EMBI), which extracts information from the electronic medical records of all ED visits and was available for each of the included EDs. The eligible patients were 18 years of age or older and met the ICD-9 criteria for migraine. Patients with stroke or intracranial hemorrhage were excluded along with those who received nitrates. In total, 1222 visits were included in the analysis [19].

The study found that opioids were used as a first-line agent in 29.5% of visits and as a rescue agent in 49.4% of visits where additional medications were required. Prevalence of opioid orders was greatest in the community ED and least in the academic ED. The study also found that patients who did not receive opioid medications had a 30.3% reduction in their length of stay. Also, in visits where opioids were prescribed, the patients had almost 40% more repeat visits during the study period. Of the non-opioid medications prescribed, metoclopramide was the most common followed by ketorolac, IV fluids, diphenhydramine, and ondansetron [19].

The authors concluded that opioids are associated with increasing repeat visits, needing to order rescue medications, and increasing length of stay. The article does not mention the doses and administration route of the medicines and it is difficult to draw conclusions about which alternative medication is superior since the study only compares opioid versus non-opioid medications [19].

## Conclusions

Effective treatment for migraine sufferers is an important goal not just for patients and their families, but for the healthcare system as a whole. Despite the increase in the number of opioid-related deaths in the United States, the general push to reduce the number of opioid prescriptions, and the various non-opioid alternatives to migraine treatment, a significant number of patients still receive opioids for the treatment of migraine in the ED.This review discussed a number of studies that not only demonstrated that the dopaminergic antagonists prochlorperazine and metoclopramide are superior to opioids and some other alternatives in the treatment of acute migraine in the emergency setting, but also suggest that opioids may actually lead longer and more frequent ED visits.The future of understanding migraine management lies in elucidating the long-term effects of treating acute migraine headache with dopaminergic antagonists. Additionally, further research is necessary to assess the efficacy of dopaminergic antagonists in comparison to other types of opioids. While each of the studies in this review did have certain limitations, collectively they suggest that patients seen in the ED for migraine headache should be given metoclopramide or prochlorperazine as first-line abortive agents in order to achieve sustained patient relief and improved emergency department efficiency.
